# CRISPR/Cas9-mediated TGFβRII disruption enhances anti-tumor efficacy of human chimeric antigen receptor T cells in vitro

**DOI:** 10.1186/s12967-021-03146-0

**Published:** 2021-11-27

**Authors:** Khadijeh Alishah, Matthias Birtel, Elham Masoumi, Leila Jafarzadeh, Hamid Reza Mirzaee, Jamshid Hadjati, Ralf-Holger Voss, Mustafa Diken, Sedighe Asad

**Affiliations:** 1 Department of Biotechnology, College of Science, University of Tehran, Tehran, Iran; 2 TRON-Translational Oncology at the University Medical Center of Johannes Gutenberg University gGmbH, Mainz, Germany; 3 Department of Immunology, School of Medicine, Tehran University of Medical Science, Tehran, Iran; 4 Department of Research Center for Immunotherapy (FZI), University Medical Center (UMC) of the Johannes Gutenberg University, Mainz, Germany

**Keywords:** CAR T-cell therapy, Coinhibitory T-cell signaling, TGFβ receptor II, Genome editing, CRISPR/Cas9 knockout, IVT-RNA

## Abstract

**Background:**

CAR T-cell therapy has been recently unveiled as one of the most promising cancer therapies in hematological malignancies. However, solid tumors mount a profound line of defense to escape immunosurveillance by CAR T-cells. Among them, cytokines with an inhibitory impact on the immune system such as IL-10 and TGFβ are of great importance: TGFβ is a pleiotropic cytokine, which potently suppresses the immune system and is secreted by a couple of TME resident and tumor cells.

**Methods:**

In this study, we hypothesized that knocking out the TGFβ receptor II gene, could improve CAR T-cell functions in vitro and in vivo. Hereby, we used the CRISPR/Cas9 system, to knockout the TGFβRII gene in T-cells and could monitor the efficient gene knock out by genome analysis techniques. Next, Mesothelin or Claudin 6 specific CAR constructs were overexpressed via IVT-RNA electroporation or retroviral transduction and the poly-functionality of these TGFβRII KO CAR T-cells in terms of proliferation, cytokine secretion and cytotoxicity were assessed and compared with parental CAR T-cells.

**Results:**

Our experiments demonstrated that TGFβRII KO CAR T-cells fully retained their capabilities in killing tumor antigen positive target cells and more intriguingly, could resist the anti-proliferative effect of exogenous TGFβ in vitro outperforming wild type CAR T-cells. Noteworthy, no antigen or growth factor-independent proliferation of these TGFβRII KO CAR T-cells has been recorded. TGFβRII KO CAR T-cells also resisted the suppressive effect of induced regulatory T-cells in vitro to a larger extent. Repetitive antigen stimulation demonstrated that these TGFβRII KO CAR T-cells will experience less activation induced exhaustion in comparison to the WT counterpart.

**Conclusion:**

The TGFβRII KO approach may become an indispensable tool in immunotherapy of solid tumors, as it may surmount one of the key negative regulatory signaling pathways in T-cells.

**Supplementary Information:**

The online version contains supplementary material available at 10.1186/s12967-021-03146-0.

## Background

Adoptive cell transfer of antigen receptor modified T-cells is a promising new tool for the treatment of cancer. Besides the modification of T-cells with tumor specific T-cell receptors (TCRs), chimeric antigen receptor (CAR)-based therapies have come into focus for the treatment of several different tumor entities. CAR T-cell therapy can be regarded as one of the most promising cancer therapies in refractory hematological malignancies. The innovative idea of combining efficient, sensitive and HLA-independent antigen recognition properties of monoclonal antibodies and killing mechanism of T-cells made this system extraordinary in tracing and eradicating tumor cells [1], which has been clinically approved in patients with ‘liquid’ tumors such as B cell malignancies [2, 3]. However, the other class of malignancies, solid tumors, have a multi-faceted line of strategies to escape killing by CAR T-cells, these include but are not restricted to lack of tumor specific antigens on tumor cells, imposing hurdles in trafficking and penetration of T-cells into encapsulated tumors, which on top are characterized by a hostile, immunosuppressive tumor microenvironment (TME) along with intrinsic counter-regulatory mechanisms of T-cells [4].

TGFβ is one of the most critical regulators in the TME, and known to be secreted by many cancer cells such as ovarian cancer cells or by components of the immune system and stromal cells [5]. This cytokine is a pleiotropic cytokine which can modulate processes such as cell invasion, immune regulation governing suppression of host immune surveillance and regulatory T-cell differentiation, and microenvironment modification, altogether leading to tumor progression [6, 7]. Accordingly, inhibiting TGFβ signaling has the potential to counteract the immunosuppressive milieu and boost CAR T-cell effector functions. Upon binding of TGFβ to TGβRII, TGβRI is recruited, phosphorylated, and activated to phosphorylate the downstream mediators, Smad2 and Smad3, which further coupled with Smad4, the latter which subsequently translocate to the nucleus, and modulates transcription of several genes such as those involved in apoptosis, extracellular matrix neogenesis and immunosuppression [8]. As TGFβRII is the initiating receptor in the TGFβ signaling cascade and isoforms are not detected yet, it was more reasonable to target it rather than TGFβRI. It has been shown that inhibiting TGFβ signaling via overexpressing dominant-negative TGFβRII can actively improve the antitumor functions of T-cells and CAR T-cells in a competitive manner to WT counterpart [9, 10]. Moreover, a clinical trial (ClinicalTrials.gov: NCT03089203) testing the safety and efficacy of the dn TGFβRII in prostate specific membrane antigen (PSMA)-specific CAR T-cells, recently reported on relapsed and refractory metastatic prostate cancer [11]. All these data suggest a potentially methodical improvement by using CRISPR/Cas9 technology to knock out endogenous TGFβRII expression to generate engineered TGFβ-resistant CAR T-cells to cope with the immunosuppressive environment within the tumor.

To address CRISPR/Cas9-driven TGFβRII KO CAR T-cell therapy in solid tumors, this study entailed two cancer associated antigens which are not or rarely expressed on normal tissues. Claudin 6 (CLDN6) is an oncofetal tight junction protein which is frequently expressed on various human solid cancers such as ovarian cancer cells [12, 13]. The other tumor antigen is taken from mesothelin (MSLN), a glycosylphosphatidylinositol-anchored cell surface protein that is expressed at low levels on normal mesothelial cells [14]. However, it is vastly overexpressed on a wide range of cancer tissues of epithelial origin including ovarian cancer [15].

In the current study, we hypothesized that the combination of a MSLN or CLDN6 CAR specific T-cells and a knockout of TGFβRII will unleash CAR T-cell functions even under immunosuppressive conditions applied in vitro. We could successfully eliminate expression of the TGFβ receptor II in human CD4 and CD8 T-cells using the CRISPR/Cas9 genome editing system. After introducing the CAR coding region via electroporation of in vitro transcribed RNA (IVT RNA) or retroviral transduction into the TGFβRII KO T-cells, these engineered TGFβRII KO CAR T-cells outperformed WT CAR T-cells by means of proliferation, cytotoxicity and cytokine secretion in the presence of exogenous TGFβ. Moreover, they were also able to resist the immunosuppressive effect of autologous iTregs more robustly in comparison to WT CAR T-cells. They also demonstrated improved function and less exhaustion after multiple rounds of antigen stimulation and showed no activation induced exhaustion, which eventually, ended up with better cytotoxicity after second or third antigen exposures.

## Material and methods

### Cell lines and culture conditions

Human cell lines, including ovarian cancer NIH-OVCAR-3 (RPMI + 20% FBS), SK-OV-3 (McCoy’s 5a + 10% FBS), cervical cancer HeLa (EMEM + 10%FBS), lung cancer COLO-699 N (RPMI + 10% FBS), breast cancer c.l. MDA-MB-231 (RPMI + 10% FBS), teratocarcinoma c.l. PA1-SC12 (MEM + 10%FBS) and HEK-293 embryonic kidney cells (DMEM + 10%FBS) have been used in this study. MDA-MB-231 and its lentiviral derivative MDA-MB-231-hCLDN6 (DMEM + 10%FBS) were also used in some experiments. The cells were routinely checked for mycoplasma contamination. All cell lines were from ATCC except COLO-699 N which was from ECACC.

### Antibodies

CAR Mesothelin surface expression on human T-cells was assessed using primary antibody goat anti-human IgG F (ab’)2 Biotin (BioRad, Hercules, CA) along with secondary conjugate Streptavidin APC (BioRad). The following antibodies were also used in FACS buffer in this study: anti-PD1-PE (Biolegends 329906), anti-CCR7-Alexa-fluor 647 (BD 560921), anti-CD8-FITC (BC A07756), anti-CD8-BV421 (BD 562428), anti-HLA-A2-FITC (BD 551,285), anti-CD86-PerCp-Cy5.5 (BD 561129), anti-CLDN6-Dylight 650 (IMAB027), anti-idiotype-IMAB027- Alexa-fluor 647, anti-TIM3-APC (Biolegends 345011) and 7AAD (BC A07704). Mesothelin expression was detected using the PE conjugated anti mesothelin antibody (R&D system FAB32652P). Acquisition and analysis of all samples were performed on a BD FACS CantoI/II (BD Biosciences, San Jose, California) and FlowJo software (v7.6.1).

### T-cell isolation and activation and human iDC generation

PBMCs were isolated from buffy coats from healthy donors (blood transfusion center at University Medical Center (UMC) Mainz, Germany) by Ficoll®-Hypaque (GE Healthcare) density gradient centrifugation. Monocytes were isolated using anti-CD14 microbeads (Miltenyi Biotec) and CD8 and CD4 cells were isolated using anti-CD8 and anti-CD4 microbeads, respectively (Miltenyi Biotec) according to the manufacturer’s protocol. Immature DCs (iDCs) were differentiated from CD14^+^ cells in RPMI 1640 GlutaMAX™, 50 IU/mL penicillin, 50 μg/mL streptomycin, and 10% (v/v) human AB serum (one lambda) (denoted huRPMI), supplemented with 1000 IU/mL recombinant human (rh) GM-CSF and 1000 IU/mL rh IL-4 (Miltenyi Biotec) twice in 5 days. For T-cell activation, 2 × 10^6^ / ml of isolated T-cells were cultivated in 24 well tissue culture plates precoated with 2 μg/ml anti-CD3 antibody (clone OKT3; Bioxcell) in huRPMI supplemented with 100 IU/mL IL-2 for 2 to 3 days. Cells were then harvested, washed and resuspended in huRPMI with 50 IU/mL IL-2 and either rested or immediately submitted to further processing steps.

### In vitro transcription

The genetic construct encoding the MSLN specific chimeric antigen receptor (CAR) containing fully human anti MSLN scFv, CD8α hinge/transmembrane region, and 41BB and CD3ε cytosolic signaling domains was generated according to the US patent 9272002 B2 and synthesized chemically. The KOZAK/coding region was subcloned into pST1 [16]-derivative MP3 553 pST1-deltaEar1-hAg-SmaI-2hBgUTR-A30L70 which is optimized for RNA in vitro transcription concerning polyA-length and composition. The codon optimized full length ORF of human mesothelin (NP_005814.2) was also subcloned into the same vector. For in vitro transcription, 10 μg of plasmid preparations were digested with SapI to be linearized and then purified using QIAquick PCR purification kit (Qiagen). The linearized plasmids were subjected to T7 RNA polymerase reaction in the presence of all four ribonucleotides and D2 cap for 3 h at 37 °C. Subsequently, template DNA was fully digested using Turbo DNase. RNA was purified using components of RNAeasy Mini kit (Qiagen) and eluted into RNase-free water. IVT-RNA concentration was adjusted to 1 μg/μl and stored at − 80 °C. The quality and integrity of the RNAs were confirmed using 1.5% MOPS gel. IVT-RNAs encoding the second generation CAR 8BBz CLDN6 and CLDN6 were prepared same way [13].

### Design and construction of CRISPRs

Single guide RNAs (sgRNA) specific for exon 2 of TGFβRII was designed using the CRISPR design tool provided by Synthego (https://design.synthego.com/#/) and chemically synthesized as O-methyl-protected derivatives. Three highest-ranking gRNAs of top ten with lowest mismatch score from both strands of human TGFβRII (Gene ID: 7048, NCBI) were selected comprising the following sequences: gRNA-3: GAAGCCACAGGAAGUCUGUG, gRNA-5: AUGAUAGUCACUGACAACAA, and gRNA-7: GCAGGAUUUCUGGUUGUCAC. The coding sequence of Cas9 was subcloned from pCAG-T3-hCAS-pA (Addgene #48625) into pST1 and IVT-RNA was prepared as described before.

### Generation of TGFβRII^−^ T-cells

T-cells were washed two times with OPTI-MEM (Invitrogen) and resuspended in serum free OPTI-MEM at a final concentration of 6–12 × 10^7^ cells/mL. Afterwards, 100 μL of cell suspension was mixed with RNAs: 10 μg of Cas9 IVT-RNA and 5 μg of gRNA 3/5/7 were electroporated in a 2-mm cuvette (VWR) using a PA-4000 (Harvard Apparatus BTX) at 400 V voltage and 2 ms single pulse length. Following electroporation, the cells were immediately transferred to prewarmed huRPMI culture media supplemented with IL2 (50 IU/mL) and incubated at 37 °C and 5% CO_2_ for 5 days.

### Antigen and CAR surface expression via IVT-RNA

Wild type or TGFβRII KO T-cells were washed two times with serum free X-vivo 15 and then resuspended in the same medium to the final concentration of 4 × 10^7^ cells/mL. Afterwards, 250 μL of the cells were mixed with 15 μg of IVT-RNA CAR MSLN or 10 μg of IVT-RNA CAR CLDN6 in a 4-mm cuvette (VWR) using a PA-4000 (Harvard Apparatus BTX) at 495 V and 9 ms. Following electroporation, the cells were immediately transferred to prewarmed culture media and incubated at 37 °C and 5% CO_2_ for 20 h prior to functional assays. iDCs were treated the same before electroporation and then adjusted to a cell density of 2 × 10^7^ cells/mL Cells were then electroporated at 300 V for 12 ms with 2 μg IVT-RNA CLDN6 or 10 μg MSLN. For luciferase based cytotoxicity assay the target cells were co-electroporated with 5 μg of luciferase IVT-RNA.

### Transduction and CRISPR/Cas9 engineering of the human T-cells

For long term cytotoxicity assay, it was necessary to stably express CAR constructs in T-cells. MACS-isolated human CD8^+^ from fresh PBMCs were pre-activated by Dynabeads™ Human T-Activator CD3/CD28 (Gibco) in a beads to CD3 + T-cell ratio of 1:1, in the presence of 100 IU/ml IL-2. 48 h after bead-activation, cells were seeded and incubated on RetroNectin (Takarabio) pre-coated plates (200 μl of 20 μg/ml) saturated with CAR gamma-retroviral supernatants by centrifugation (30 min, 15 °C, 1500 rpm) to obtain an multiplicity of infection (MOI) for T-cell: viral particle ratio of 1. After extra 48 h, pre-activation Dynabeads™ Human T-Activator CD3/CD28 were removed from culture and cells were electroporated for Cas9 mRNA and TGFβRII-specific gRNAs as described before. Non-transduced T-cells served as controls. Heterologous CAR expression on transduced human T-cells was assessed via flow cytometry.

Platinum-E cells were used for preparation of GALV pseudotyped viral particles. Cells were transfected with TransIT-LT1 (Mirus) according to the manufacturer’s instructions. Retroviral supernatants were collected 48 and 72 h after transfection and titers were evaluated using Jurkat cells.

### Gene disruption efficiency assay using T7 endonuclease I assay, sequencing and TIDE assay

Five days after electroporation with gRNA and Cas9 mRNA, genomic DNA was extracted from the cells using QIAmp DNA mini kit (Qiagen) according to the manufacturer’s instructions. The genomic regions flanking the sgRNA target site in the TGFβRII gene were PCR amplified with the following primers: TGFβRII forward: 5’-AGAAAGTGGACCTTATGACAACCA, and TGFβRII-reverse: 5’-AGGAGGTGTCGGTTAAATGACTAC using Q5 high fidelity DNA polymerase (NEB). The PCR products were either purified and Sanger sequenced with TGFβRII-sequencing primer: 5’-TCTGATGTGAAGGAATTATTTTGCCT and then subjected to TIDE analysis using the online tool http://tide.nki.nl (Analyses were performed using a reference sequence from a Cas9 mock-electroporated sample) or subjected to T7EI Nuclease (NEB) assay as previously described. Briefly, the PCR products were first heated up to 90 °C to be denatured and then the temperature gradually decreased to 10 °C to reanneal the product. The mixtures were then subjected to T7E1 digestion for 15 min which recognizes mismatches, and finally analysed on a 2% agarose gel.

### Luciferase, spheroid and impedance-based cell lysis assays

For luciferase-based cytotoxicity assay, WT CAR T-cells or TGFβRII KO CAR T-cells were cocultured in triplicates with the Ag and luciferase expressing target cells at the indicated E:T ratio 20 h after electroporation in white Nunclon Delta surface 96 well plates. Three hours after setting up the coculture, 50 μl of the substrate solution, 1 mg/ml Luciferin (BD, moonlight D-luciferin) and 50 mM HEPES, was added to each well. Triton-X100 (0.25% f.c.) was added for full lysis control. The bioluminescence emission was recorded using Tecan Infinite 200 reader at indicated time points and the cytotoxicity was calculated as follows:

$$specific lysis\left(\%\right)=\left(1-\frac{({L}_{sample}-{L}_{max})}{({L}_{min}-{L}_{max})} \right)*100$$

${L}_{sample}$: Luminescence mean by sample lysis

${L}_{max}$: Luminescence mean by maximal lysis

${L}_{min}$: Luminescence mean by spontaneous lysis

To mimic the 3D nature of tumors, spheroid-based cytotoxicity assay was performed using the Incucyte live cell imaging system. For generating the spheroids, tumor cell lines were electroporated with eGFP mRNA and seeded in spheroid plates (Corning #7007) for 24 h. After 24 h, the generations of spheroids and fluorescence emissions were confirmed using the Incucyte, and WT CAR T-cells or TGFβRII KO CAR T-cells were added to the corresponding wells, and reduction of the fluorescence intensity was monitored over time. The data were analyzed using Incucyte software and the background was subtracted using the Top Hat algorithm.

An impedance‐based tumor lysis cell culture system (xCELLigence, ACEA Biosciences) was used to assess the long-term T‐cell cytotoxicity in real-time. By means of an electrode‐bottomed plate, the device measures the electrode impedance of adherent target cells which corresponds to cell viability, and reports it using a unitless parameter called Cell Index (CI). Cell lysis leads to detachment of target cells decreasing CI over time. Target cells were seeded on 96‐well electrode-bottomed microtiter plates (E-Plate® 96) at 2 × 10^4^ cells/100 μL/well. After 20 to 24 h, right before when CI starts to plateau, the effector cells were added at indicated E:T ratios and the CI was recorded every 15 min. To correct for diverging growth curve properties on a 96-well plate (‘edge effects’), CIs of any time point were first normalized to the CI (NCI) shortly before effector cells were added to every well and averaged over replicates. Specific lysis was calculated via the following formula taken from RTCA software:

$$specific lysis\left(\%\right)=\left(1-\frac{({NCI}_{sample}-N{CI}_{max})}{(N{CI}_{min}-N{CI}_{max})} \right)*100$$

${NCI}_{sample}$: Normalized cell index mean by sample lysis

${NCI}_{max}$: Normalized cell index mean by maximal lysis

${NCI}_{min}$: Normalized cell index mean by spontaneous lysis

### Repetitive antigen stimulation assay

Target cells were seeded on Xcelligence plates (2 × 10^4^ cells/100 μL/well), and after 20 to 24 h, WT or TGFβRII KO CD8 + T-cells, which were retrovirally transduced to express a CLDN6 specific CAR were added at an E:T ratio of 5:1 in the presence or absence of TGFβ. Cytotoxicity was monitored for 48 h in real time. Subsequently, CAR T-cells were collected from wells, washed, counted again and added to a new Xcelligence plate containing previously seeded target cells the same way as before. The amount of cell lysis was monitored again for 48 h, and the same procedure repeated one more time. The concentration of TGF-β1 was maintained at 5 ng/mL in the treatment group.

### Proliferation

Either WT CAR T-cells or TGFβRII KO CAR T-cells were labeled with 0.8 μM carboxyfluorescein diacetate succinimidyl ester (CFSE, Invitrogen) or 10 µM eBioscience™ Cell Proliferation Dye eFluor™ 450 (CPD450) and cocultured with Ag mRNA-electroporated iDCs at an E:T ratio of 5:1 in round-bottom 96 well plates with different doses of TGF-β1 (carrier-free; R&D Systems). An aliquot of culture supernatants was harvested after 24 h for cytokine analysis.

After 5 days of coculture T-cells were stained with anti-CD8 or anti-CD4 antibody and the reduction of CFSE-staining as readout for cell divisions measured using the high through put sampler (HTS) implemented on a FACS canto II device.

### Cytokine enzyme-linked immunosorbent assay (ELISA)

WT CAR T-cells or TGFβRII KO CAR T-cells were cocultured with Ag mRNA-electroporated iDCs or cancer cell lines at different E:T ratios in round-bottom 96 well plates in a final volume of 200 μl. After 24 h, 100 μl of supernatant was collected and frozen down to − 80 °C for analysis later on, or 1:2–1:10 dilutions in assay diluent were assayed immediately by incubating for 3 h at 37 °C on high protein binding 96-well plates precoated with a cytokine-specific capture antibody. After repetitive washing immobilized cytokines (IFN-γ) were detected with a biotinylated detection antibody/streptavidin-HRP-conjugate after addition of the substrate NBT. Color development was photometrically quantified on TECAN Sunrise ELISA reader system and quantified by calculation of μg/ml via a cytokine standard developed in parallel. The supernatants were assayed for producing human IL-2 and IFN-γ using human ELISA kit (Invitrogen), while Granzyme B (GrB), TNF-α and GM-CSF were quantified by the DuoSet ELISA Development Kit (R&D Systems).

### iTreg generation in vitro suppression assay

CD4^+^ T-cells were positively selected from fresh PBMCs using CD4 microbeads (Miltenyi-Biotec) according to the manufacturer’s protocol and rested for 3 h. Cells were then plated in 24 well plates precoated with 5 μg/ml anti-CD3 antibody (clone OKT3; Bioxcell) at 2 × 10^6^ cells/well in serum-free X-Vivo 15 medium (Lonza) supplemented with 1 μg/ml soluble anti-CD28 antibody (Biolegend, ultra-LEAF grade) and 100 IU/ml IL-2. Cells stimulated with only these reagents served as control. For iTreg generation, 5 ng/ml of TGF-β1 (carrier-free; R&D Systems), 10 nM of all trans retinoic acid (ATRA) (Sigma-Aldrich) and 100 ng/ml of rapamycin (Calbiochem EMD Millipore) were added to the culture media. Cells were incubated for 6 days at 37 °C/5% CO_2_ in an incubator. Thereafter, CD4 + T-cells being committed to differentiate into induced Tregs (iTregs) were washed and rested for two to three days in X-Vivo 15 medium with 50 U/ml IL-2. For setting up the suppression assay, iTregs were washed again and resuspended in fresh medium without any IL-2. The responder (Tresp) cells were either WT CAR T-cells or TGFβRII KO CAR T-cells. Tresp were labeled with 10 μM CPD-450 as described before and the coculture set up as followed: In each well of round-bottom 96 plate, constant number of Tresp cells (5 × 10^4^) and Ag expressing iDCs as target cells (1 × 10^4^) were coseeded and additionally, cocultivated with suppressive iTregs or activated CD4 + T-cells as negative control at different ratios (2:1,1:1, 0.5:1 and 0.25:1). Cells were cultured for 3 to 4 days and suppression in proliferation was analysed by flow cytometry.

## Results and discussion

### MSLN and CLDN6 specific CAR T-cells recognize and kill cognate antigen expressing tumor cell lines

**Fig. 1:**
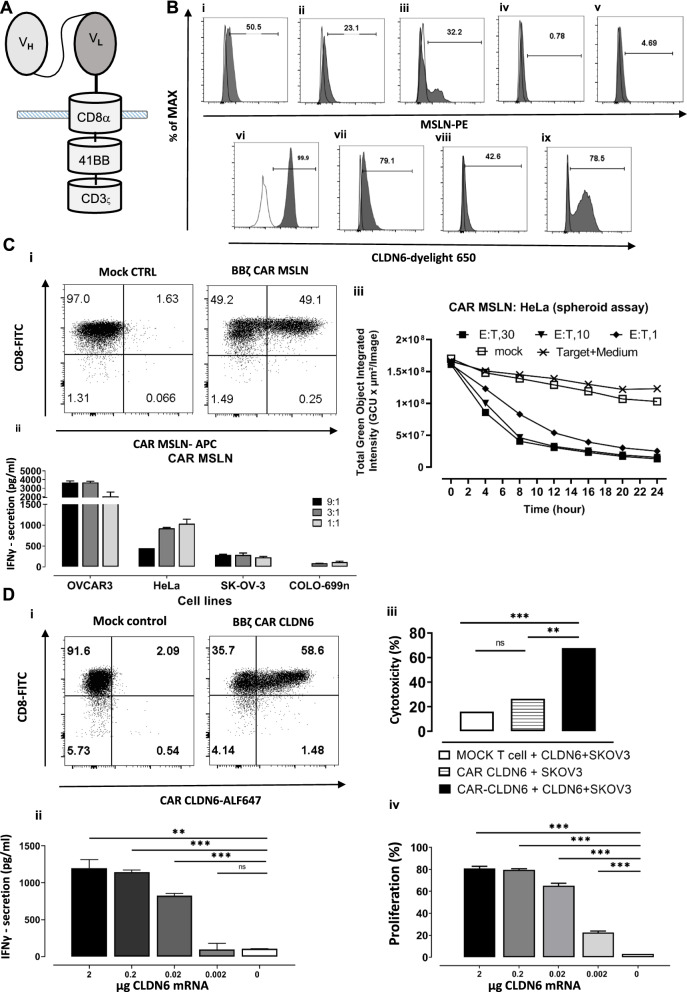
MSLN and CLDN6 are highly expressed on tumor cell lines and CAR CLDN6 or MSLN recognize and kill them efficiently. **A** A 2nd Gen. CAR CLDN6 or MSLN construct based on chimerisation to the hinge, transmembrane, and cytosolic region of CD8α, the cytosolic region of 4-1BB and CD3ς for costimulatory and stimulatory signaling, respectively. **B** MSLN expression on i) Hela and ii) OVCAR-3 cell line, iii) exogenous MSLN expression on iDCs after electroporation of 10 μg MSLN IVT-RNA, and non-expressing tumor cell line iv) SK-OV-3 and v) Colo-699 N. Endogenous CLDN6 expression on vi) PA-1 and vii) OVCAR-3 cell surface viii) exogenous CLDN6 expression on MDA-MB-231 after transduction with CLDN6 encoding virus particles, and ix) CLDN6 expression on iDCs after electroporation of 2 μg CLDN6 IVT-RNA, respectively. **C**: i) CAR MSLN expression on human CD8 T cells one day after electroporation with 10 μg CAR MSLN IVT-RNA, ii) IFN-γ secretion assay proved that MSLN-specific CAR recognizes MSLN positive cells (HeLa and OVCAR-3), while they do not react with antigen negative cell lines (SK-OV-3 and COLO-699 N), iii) Spheroid killing assay demonstrated that CAR MSLN T-cells recognize and kill HeLa tumor spheroids at also a very low E:T-ratio. **D**: i) CAR CLDN6 expression on human CD8 T cells one day after electroporation with 10 μg CAR CLDN6 IVT-RNA, ii) IFN-γ secretion assay proved that CAR CLDN6 can specifically recognize CLDN6 electroporated iDCs in a dose dependent manner while they do not react with antigen negative cells, iii) Xcelligence based killing assay showed that CAR CLDN6 T-cells recognize and kill exogenously CLDN6 expressing SK-OV-3 cell lines, while mock T-cells remained inactive. vi) CFSE based proliferation assay showed that CAR CLDN6 T cells divided strongly up to a frequency of 80%, not only in the presence of a high dose of heterologously expressed CLDN6 in iDCs (2 μg), but also at a dose of as low as 0.02 ug CLDN6. P values were determined by one-way Anova using multiple comparison test. *P < 0.05; **P < 0.01; ***P < 0.001; ****P < 0.0001. In all experiments, mean ± SD of three technical replicates are given and experiments, involving T cells, are repeated for at least three donors

In this study, we focused on CAR T-cells specific for the tumor antigens mesothelin and claudin 6 (Fig. 1A), which are highly expressed on tumor cells but rarely detected on normal cells. We either used antigen overexpressing cell lines, HeLa (Fig. 1B i) and OVCAR-3 (Fig. 1B ii) for MSLN-specific antigenicity, PA-1 SC12 (Fig. 1B vi) and OVCAR-3 (Fig. 1B vii) for CLDN6-reactivity or exogenously overexpressed the antigen on the cell surface of iDCs (MSLN and CLDN6) (Fig. 1B iii, ix) or (MDA-MB-231) (Fig. 1B viii) via IVT-RNA electroporation or virus transduction. The MSLN-negative cancer cell lines SK-OV-3 (Fig. 1B iv) and Colo-699 N (Fig. 1B v) are also used in some experiments.

We activated CD4 + /CD8 + T-cells and transfected them with MSLN (15 μg) and CLDN6 (10 μg) CAR encoding IVT RNAs. At this high dose, we could monitor around 50% CAR expression for both antigen specificities (Fig. 1C i, D i).

To confirm that the CAR MSLN T-cells are functional in vitro we performed multiple assessments. In the first step, IFN-γ secretion was examined against different-cell lines: CAR MSLN T-cells secreted high level of IFN-γ towards MSLN expressing cell lines, up to 4000 pg/mL in case of OVCAR-3 and 1500 pg/mL in case of HeLa cell line as target cell (Fig. 1C ii). There was no significant secretion of this cytokine against the negative control cell lines SK-OV-3 and COLO-699 N. Furthermore, cell cytotoxicity was evaluated by means of a 3D culture spheroid lysis assay (Fig. 1C iii). Solid phase spheroid cells reflect the in vivo situation in some aspects such as actively dividing tumor cells at its boundaries with optimal nutrients provision, and at its core tumor cells becoming apoptotic or necrotic [17]. However, it does not embrace immunosuppressive and angiogenetic effects in TME. Our data confirmed that the CAR MSLN T-cells successfully kill the tumor spheroids even at a low E:T ratio of 1:1, which could achieve almost 100% killing in 24 h as opposed to mock T-cells. Decrease of fluorescence in negative controls reflects the degradation of GFP encoding mRNA over time. Conclusively, these data proved cytokine secretion and cytotoxic function of CAR MSLN T-cells. We also performed this experiment for the MSLN-negative cell line SK-OV-3 and data showed that the CAR MSLN T cells did not exert any cytotoxicity on this cell line at a high E: T ratio of 30:1. The recorded slow decrease in green intensity in both mock and CAR MSLN T cells is probably due to the half-life of the electroporated mRNA (Additional file 1: Fig. S1).

To assess CAR CLDN6 functionality, CAR CLDN6 T-cells were cocultured with IVT-RNA electroporated iDCs expressing CLDN6 heterologously at increasing doses for an E:T ratio of 5:1 to quantify secreted IFN-γ in an ELISA. The amount of detected cytokine ranged between 1000 pg/ml for 2 μg electroporated CLDN6 and 100 pg/ml for 0.02 μg electroporated CLDN6 (Fig. 1D ii), and hence, decreased dose-dependently.

For the impedance based cytotoxicity assay, we electroporated the SK-OV-3 cell line with 2 μg CLDN6 RNA and seeded them on xcelligence plates. After 24 h, the CAR CLDN6 or the mock T-cells were added at an E:T ratio of 5:1 and the impedance changes were recorded over time. Successful killing of CLDN6^+^ SK-OV-3 cells by CAR CLDN6 effector T-cells (70%) was confirmed, while no relevant cytotoxicity was observed for mock T-cells (Fig. 1D iii).

We also evaluated the proliferation of CAR CLDN6 T-cells applying a CFSE dilution-based assay in a dose dependent manner. These CAR T-cells divided strongly up to a frequency of 80%, not only in the presence of a high dose of heterologously expressed CLDN6 in iDCs (2 µg), but also at a dose of  as low as 0.02 µg CLDN6 (70%; Fig. 1D iv). Notably, no proliferation was detectable in the presence of mock-electroporated iDCs. In conclusion, our data confirmed that both CARs specific for MSLN and CLDN6 elicited superior effector functions against antigen expressing tumor cell lines or iDCs.

### Exogenous TGFβ impairs CAR T-cell proliferation in vitro

**Fig. 2:**
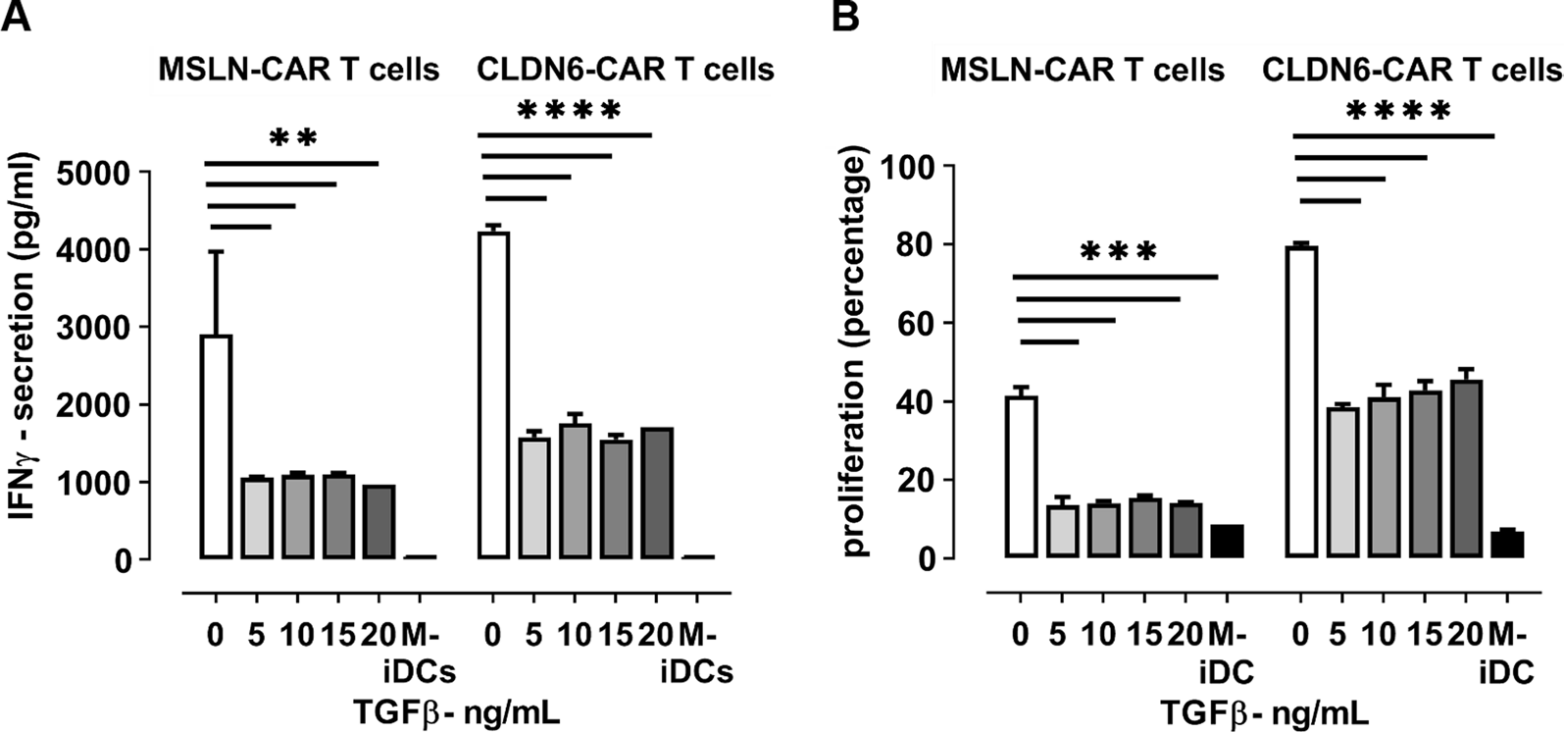
Exogenous TGFβ impairs CAR T-cell cytokine secretion and proliferation in vitro. **A** TGFβ is able to inhibit IFN-γ secretion of MSLN- or CLDN6-specific CAR 4-1BBζ T-cells towards antigen-pulsed iDCs in a broad dose range (5-20 ng/mL) and on a short time line (20 h). **B** TGFβ is also able to inhibit proliferation of MSLN- or CLDN6-specific CAR 4-1BBζ T-cells towards antigen-pulsed iDCs in a broad dose range (5-20 ng/mL) and on a long time line (5 days). P values were determined by two-way Anova using multiple comparison test. *P < 0.05; **P < 0.01; ***P < 0.001; ****P < 0.0001. In all experiments, mean ± SD of three technical replicates are given and experiments, involving T cells, are repeated for at least three donors

Due to the fact that TGFβ is one of the key regulators of the immune system in the tumor microenvironment, which vastly suppresses CAR T-cell function [9], we hypothesized that one of the hurdles in solid tumor CAR T-cell therapy arise from high levels of TGFβ in TME. To test this hypothesis, we performed a series of in vitro tests to find out if exogenous TGFβ can exert a suppressive effect on our CAR T-cells specific for 2 different tumor antigens. IVT electroporated iDCs (CLDN6/ MSLN) were cocultured with CAR CLDN6 or CAR MSLN T-cells at increasing doses of TGFβ. A broad range of exogenous TGFβ added (5–20 ng/ml) could significantly suppress CAR T-cell functions in terms of cytokine secretion (Fig. 2A) and proliferation (Fig. 2B) which is consistent with previous reports. Secretion of IFN-γ, the most prevailing immunostimulatory T-cells’ cytokine, is very susceptible to even the lowest dose of TGFβ added, dropping to less than 40% of the IFN-γ secretion in absence of TGFβ. In line to that, proliferation of both MSLN and CLDN6 specific CAR T-cells was reduced to less than of 50% of their native proliferation (i.e. absence of TGFβ) in the presence of even very low dose of TGFβ (5 ng/ml). Noteworthy, the suppressive effect of TGFβ did not only last for short-term incubation times (< 24 h) as it is demonstrated here for an IFN-γ ELISA, but was also obvious for long-term proliferation assays (5–6 days). In conclusion, these data demonstrated that TGFβ is a potent inhibitor of CAR T-cells, regardless of their antigen specificity.

We should mention that there is not any robust data on precise level of active TGFβ at tumor site. Data are mostly focused on blood which was in the range of 0.5–25 ng/mL in human plasma [18] and it is stated that the concentration of TGFβ is much higher in cancer patients in comparison to a normal control [19]. Reasonably, the concentration of active TGFβ should be higher in tumor in comparison to plasma because the tumor cells secrete TGFβ by themselves, which can be activated by a matrix metalloprotease. As others, such as Hou et al. [20], also used 5 ng/ml TGFβ in their studies, we decided to use this concentration as starting concentration. Since we are primarily interested in testing the potency of our TGFβ RII KO CAR T-cells, we primarily checked higher levels of TGFβ to make sure that our knocked-out groups can also resist higher levels of TGFβ which we speculate to prevail in TME.

### Deletion of TGFβ RII via CRISPR/Cas9 system

**Fig. 3:**
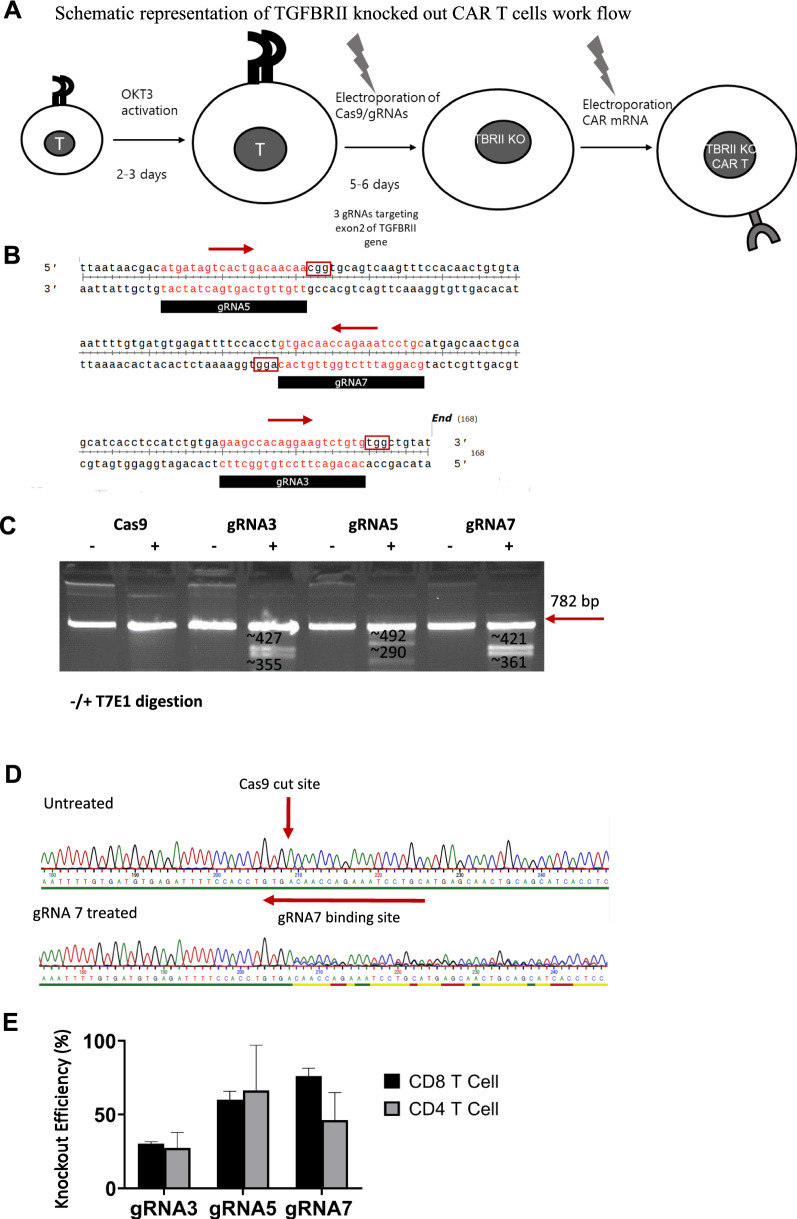
The TGFβ RII locus is successfully targeted via CRISPR/Cas9 system. **A** Schematic illustration of genomically knocking out TGFβ RII taking advantage of synthetic gRNAs targeting the TGFβRII locus and IVT-RNA of the nuclease Cas9, followed by IVT-RNA based CAR expression in human T-cells. **B** Graphic representation of the sequence and genomic location of three different gRNAs targeting exon2 of TGFβ RII and 20 bases in length, arrows indicate the polarity of the gRNAs. **C** T7 endonuclease 1 assay showing extra bands for gRNA treated T-cell groups in contrast to the control group, confirming indel formation in the gRNA treated group. **D** Histogram sequencing results for wild type and gRNA7 treated groups, elicit a heterologous sequence 4 bases inside the gRNA target target sequence upstream of complementary 5’-NGG PAM-sequence, which is in line with indel formation. **E** Bar graph of TIDE analysis illustrates that all three different gRNAs can lead to successful TGFβ RII knockout in both human CD4+ and CD8+ T-cells. In all experiments, mean ± SD of three technical replicates are given and experiments, involving T cells, are repeated for at least three donors

We prompted us to assess whether genomic disruption of TGFβRII in CAR T-cells will counteract the aforementioned functional impairment of CAR T-cells in the presence of exogenous TGFβ. To achieve this purpose, we designed a 2-step protocol comprising CRISPR/Cas9 based gene knock out of the TGFβRII locus and subsequent CAR IVT-RNA electroporation into those genomically edited primary human T-cells (Fig. 3A). The RNA-guided Cas9 nuclease from the microbial CRISPR adaptive immune system is an efficient genome editing tool which consists of nuclease Cas9 and a single-guide RNA (sgRNA) targeting a 20-bp region of the genomic region of interest [21]. We used the Synthego online tool to design sgRNAs and selected three gRNAs from the top ranked suggested sgRNAs on both strands with at least 50 nucleotides distances (Fig. 3B). The Synthego online tool uses the Doench et al. [22] scoring algorithm to select efficient sgRNAs and also ranks the sgRNAs based on the numbers of genomic off targets. Number of potential off target sites for zero, one and two mismatches was zero for gRNAs 3/5, while in the case of gRNA7, four off targets emerged with two mismatches. However, we cannot entirely rule out that off-target edits might occur which need to be assessed by next generation sequencing before entering the clinical setting. To combine the knock out procedure and CAR expression, briefly, OKT3 activated T-cells were electroporated with Cas9 mRNA alone (Cas9 control), or Cas9 and chemically synthesized O-methyl protected sgRNA targeting TGFβ RII exon 2. Five days later, TGFβ RII ablation was checked using genomic analysis techniques by means of a T7 endonuclease I assay and Sanger sequencing. Cells were subsequently electroporated with saturating amounts of IVT mRNA encoding the anti-MSLN or anti-CLDN6 CAR (Fig. 3A).

To determine the knock out efficiency, genomic DNA was extracted from the cells (Cas9 only and Cas9 with gRNAs 3, 5 or 7) and was submitted to PCR amplification using PCR primers flanking the sgRNA targeting region. An aliquot of the PCR products was subjected to T7 endonuclease I restriction and then run on an agarose gel (Fig. 3c).

T7 endonuclease I recognizes and cleaves mis-matched DNA, which could lead to extra bands on the gel in case of indel formations. The PCR product from control cells and the three gRNAs treated cells were submitted to digestion by T7E1 enzyme. While there were no extra bands in the control, all gRNA treated groups exhibited extra bands which indicates indel formations (Fig. 3C). As outlined in Fig. 3B, each gRNA targets nearby but different genomic sequences of the TGFβRII gene and hence, taking into account using identical PCR primers, the restriction pattern of T7E1 ended up with variably sized restriction products for each gRNA visible on the gel. The PCR products were also delivered for Sanger sequencing and the data were analyzed for indel frequencies applying a decomposition technique. Base ambiguities commence after base 4 of gRNA7 (5’-GTGA-A/C…), which is consistent with the postulated genomic cut site of Cas9 (Fig. 3D, Additional file 2: Fig. S2). Again, results confirmed indel formations in all gRNA treated groups (Fig. 3D/E). Although gRNA3 was prone to form less indels in comparison to the two other gRNAs (Fig. 3E), we did not exclude it from our further experiments so as to check if even a lower level of genomic TGFβRII knock out might also have a measurable effect on CAR T-cell functions. In summary, these data exhibited the successful generation of insertions/deletions (INDELs) in the TGFβ RII gene locus. We were able to demonstrate gene ablation to similar degrees for the 3 scrutinized gRNAs in both, CD8 + and CD4 + cells, respectively (Fig. 3E).

### TGFβ RII disruption in CAR T-cells enhances their in vitro effector functions

**Fig. 4:**
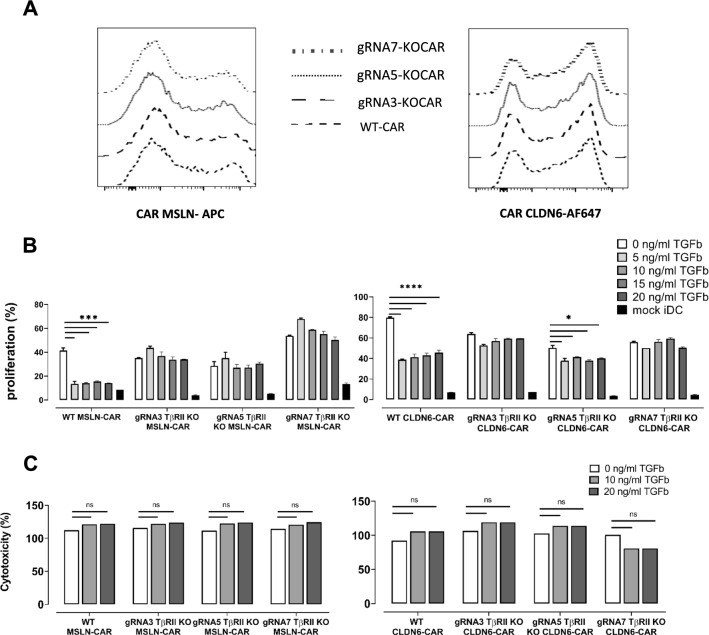
Genomic TGFβ RII disruption in CAR T-cells specific for MSLN or CLDN6 enhances their in vitro effector functions. **A** Genomic editing of TGFβRII in TGFβRII KO CAR T-cells does not impact on CAR expression irrespective of the antigen specificity when compared to WT CAR T-cells. **B** TGFβ at any dose inhibits MSLN or CLDN6 4-1BBζ CAR T-cell proliferation in the presence of antigen presenting iDCs as APCs. Of importance, proliferation of TGFβRII KO CAR T-cells remain unimpaired and stable even at a very high dose of exogenous TGFβ. Proliferation in either WT or KO groups proved to be antigen specific. **C** In short-term classical cytotoxicity assays (luciferase based) against immunostimulatory iDCs, TGFβ does not impair cytotoxicity of WT CAR or TGFβRII KO CAR T-cells. P values were determined by two-way Anova using multiple comparison test. *P < 0.05; **P < 0.01; ***P < 0.001; ****P < 0.0001. In all experiments, mean ± SD of three technical replicates are given and experiments, involving T cells, are repeated for at least three donors

**Fig. 5:**
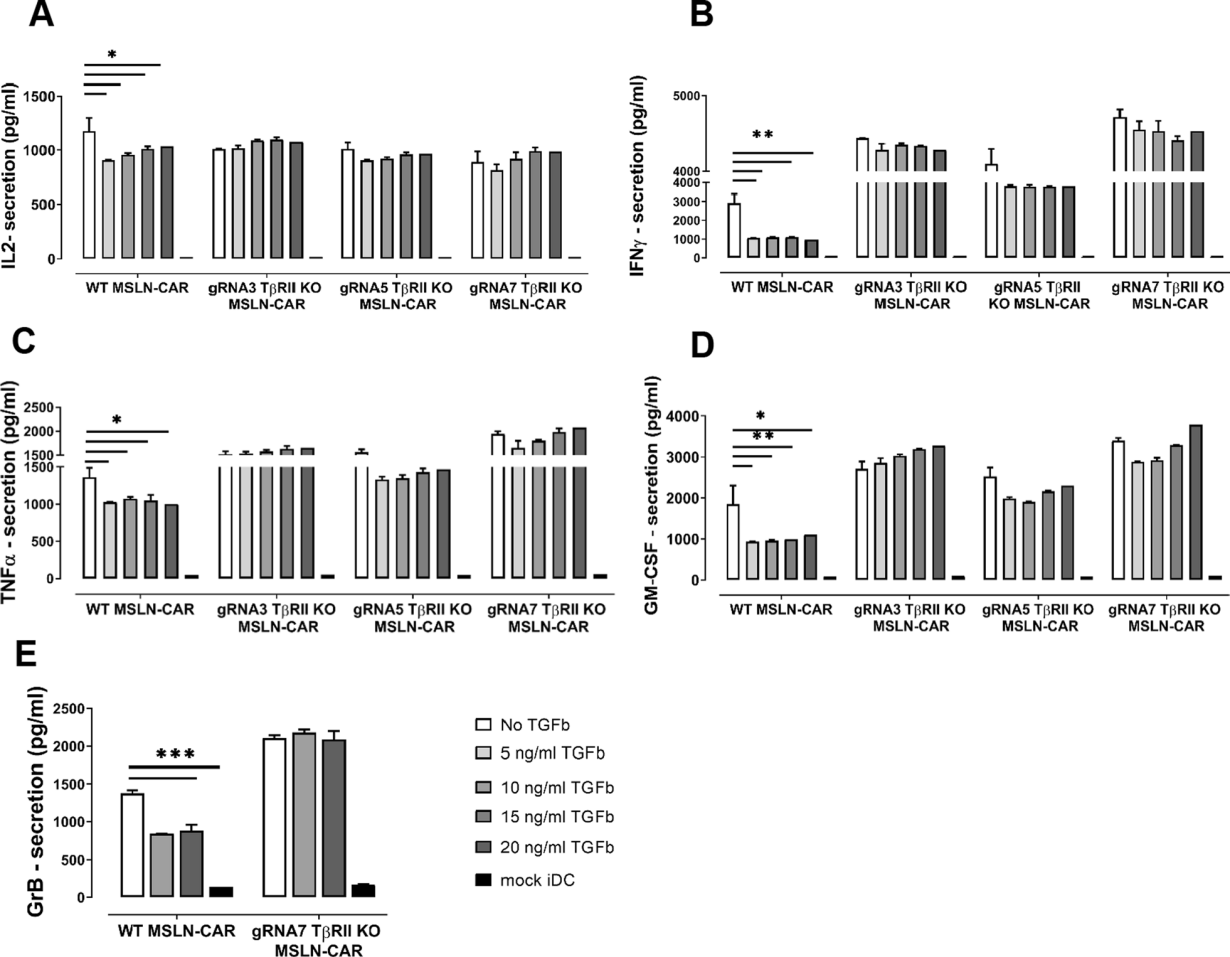
Genomic TGFβ RII disruption in CAR T-cells specific for MSLN enhances their cytokine secretion. TGFβ at any dose inhibits cytokine secretion of MSLN-specific WT CAR 4-1BBζ T-cells. **A** IL-2, **B** IFNγ, **C** TNFα, **D** GM-CSF, **E** Granzyme B, in the presence of antigen presenting iDCs as APCs. Of importance, amount of cytokines in TGFβRII KO CAR T-cells remain unimpaired and stable even at a very high dose of exogenous TGFβ. Cytokine secretion in either WT or KO groups proved to be antigen specific here as well. P values were determined by two-way Anova using multiple comparison test. *P < 0.05; **P < 0.01; ***P < 0.001; ****P < 0.0001. In all experiments, mean ± SD of three technical replicates are given and experiments, involving T cells, are repeated for at least three donors

To determine the effect of TGFβRII deficiency on CAR T-cell function, we assessed TGFβRII KO CAR MSLN and CAR CLDN6 T-cells in different assays in vitro. Due to limited KO efficacy, these experiments were done on bulk populations that contained both edited and non-edited cells. As an essential prerequisite for comparison of KO CAR T-cells with WT CAR T-cells, gene ablation of TGFβRII must not interfere with IVT-RNA-mediated CAR expression in T-cells. Indeed, we observed very similar frequencies of CAR expression on T-cells (Fig. 4A): 50% CAR expression could be observed for both, CAR-MSLN electroporated T-cells and TGFβRII KO CAR-MSLN T-cells. Similar to that, we also found up to 70% CAR expression in electroporated T-cells for CAR CLDN6 irrespective of the genomic editing step. Additionally, we analyzed proliferation of WT or KO CAR T-cells following electroporation in the absence of exogenous TGFβ to determine whether deletion of the TGFβRII gene has any impact on proliferation and/or survival of CAR T-cells. In most tests, we did not monitor any marked alterations in proliferation between WT and TGFβRII KO CAR T-cells and analysis among multiple donors did not result in a statistically elevated difference between WT and KO groups when the latter group was averaged over the 3 different gRNAs (white bars in Fig. 4B, and data not shown). Antigen independent expansion or cytokine secretion of KO CAR T-cells was absent leading to the conclusion that deletion of the TGFβRII did not result in immortality (black bars in Figs. 4B and 5).

Again, TGFβ induced reduction of cell proliferation by more than a factor of 2 in WT CAR T-cells. Intriguingly, proliferation rates were restored in CD8+ and CD4+ TGFβRII KO CAR MSLN or CAR CLDN6 T-cells following stimulation with antigen presenting iDCs even in the presence of escalating doses of exogenous TGFβ (Fig. 4B). Of note, for TGFβRII KO CAR MSLN T-cells we still observed a small but steady decline of dose rate-dependent proliferation for 2 out of 3 gRNAs tested. Beside the dose effect, this may also result from a higher fraction of non-edited CAR T-cells in bulk CD8+ T-cells used in the assay here, the former which is still susceptible to TGFβ. It is important to note that rescue of proliferation is not complete: Despite the high recovery of proliferation in TGFbRII KO CAR-T groups, we observed proliferation efficacies for KO groups in the range of only up to approx. 60%, that means at least 40% of them, either the WT fraction or even the KO fraction in bulk edited/non-edited KO groups, did still not proliferate. We also assessed the cytolytic potential of KO TGFβRII CAR T-cells specific for MSLN or CLDN6 against antigen loaded iDCs (Fig. 4C) in a bioluminescence cytolysis assay. However, in contrast to the proliferation and cytokine secretion results discussed before the killing efficiency of these KO CAR T-cells was not affected compared with the WT control. This has also been observed by others in a conventional cytotoxicity assay [23].

Nonetheless, when compared to WT CAR-T-cells, TGFβRII KO CAR-T-cells released equal amounts or a bit more of IL-2 (Fig. 5A, Additional file 3: S3A), IFN-γ (Fig. 5B, Additional file 3: S3B), TNFα (Fig. 5C, Additional file 3: S3C), GM-CSF (Fig. 5D, Additional file 3: S3D)-cytokines, and GranB (Fig. 5E, Additional file 3: S3E) (tested just for one KO group/gRNA7) when they were cocultured with iDCs expressing the cognate antigen in the absence of exogenous TGFβ. This cytokine release was dependent on CAR expression and non-electroporated T-cells (mock) failed to release cytokines in the presence of antigen positive cells (data not shown) and there was no cytokine secretion in coculture of all CAR T cell groups with antigen negative iDCs (Fig. 5, black bars). Importantly, WT CAR-T-cells lost their ability to secrete cytokines in the presence of exogenous TGFβ to varying extents (Fig. 5, Additional file 3: Fig. S3) in the order IFN-γ > GM-CSF > GranB > TNF-α > IL-2 in a TGFβ dose-independent manner. Contrary to that, TGFβRII KO CAR MSLN or CAR CLDN6 T-cells reset the immunusuppressive effect of TGFβ on cytokine secretion almost completely irrespective of the amount of suppression observed for WT CAR T-cells. Secondly, the antagonizing effect of TGFβRII KO CAR MSLN or CAR CLDN6 T-cells was on the whole independent of the dose of exogenous TGFβ added. We postulate that in general, a larger fraction of edited CAR T-cells being present in the bulk population dominate the fraction of non-edited CAR T-cells in the net outcome of function. This means to expect rather a gradual decrease of cytokine secretion in TGFβRII KO CAR T-cells, which is true for most sample groups. However, a steady TGFβ dose rate-dependent increase of GM-CSF and TNF-α secretion became only evident for KO TGFβRII CAR MSLN T-cells. This may be here due to the ratio of edited versus non-edited T-cells, the sensitivity of these cytokine secretion pathways towards TGFβ, and the somewhat lower efficacy of CAR MSLN compared with CAR CLDN6.

In total, these results suggest that TGFβ RII disruption can antagonize in all three KO groups diverse long-term and short-term functional defects induced in WT MSLN or CLDN6 4-1BBζ CAR T-cells treated with exogenous TGFβ. However, our experiments did not reveal any effect of exogenous TGFβ in short term killing assays (4 h) against immunostimulatory target cells such as iDCs, neither for WT nor KO CAR T-cells.

### TGFβ receptor II disruption augments long term cytotoxicity

**Fig. 6:**
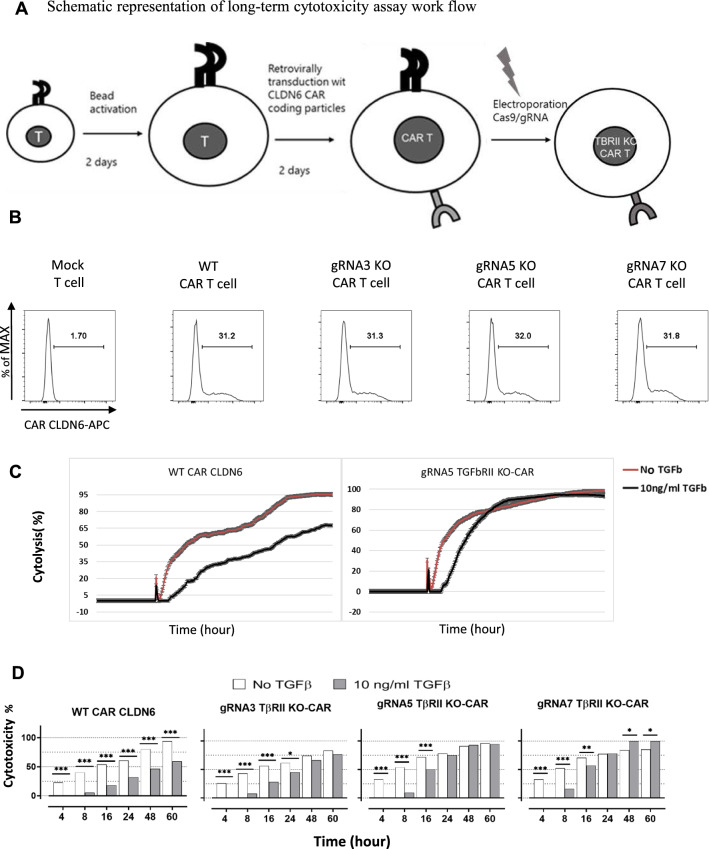
Genomic deletion of TGFβ receptor II restores long term cytotoxicity of TGFβRII KO CAR T-cells. **A** Schematic representation of long-term cytotoxicity assay work flow. Retroviral transduction of CD8+ T-cells with CAR CLDN6 supernatants is followed by CRISPR/Cas9 treatment to disrupt TGFβRII expression. **B** Equal CAR CLDN6 surface expression on retrovirally transduced WT and TGFβRII KO T-cells. **C** Impedance based cytotoxicity assay in the absence (red) and presence (black) of TGFβ. The averaged cell indexes of triplicates were normalized to the time shortly before seeding effector CAR T-cells, and then recalculated to %cytolysis according to the formula given in Material and methods. In the presence of TGFβ, WT CAR T-cells were compromised in cytotoxicity towards CLDN6+ tumor c.l. PA-1 lower the entire incubation time (> 3 days), while cytotoxicity of TGFβRII KO CAR T-cells recovered after 20 h. **D** Cytotoxicity bar chart of WT and TGFβRII KO CAR T-cells in the absence (white bars) and presence (grey bars) of 10 ng/ml TGFβ at designated time points taken from **B**. The initial gain in cytotoxicity for non-TGFβ treated KO CAR T-cells in comparison to the TGFβ-treated KO CAR T-cells levelled out over time (24 h) and led to comparable or even better cytolyses compared to WT CAR T-cells. P values were determined by two-way Anova using multiple comparison test. *P < 0.05; **P < 0.01; ***P < 0.001; ****P < 0.0001. In all experiments, mean ± SD of three technical replicates are given and experiments, involving T cells, are repeated for at least three donors

Several reports showed an inhibitory effect of TGFβ on the cytotoxicity of T-cells and CAR T-cells cells [24–27]. However, we could not reproduce any suppression in short term cytotoxicity assays against iDCs. Hence, we addressed the question whether then on a long term exposure, TGFβ can hinder the killing function of the CAR T-cells taking into account in particular the chronic prevalence of a suppressive milieu in TME over time in patients. For this, we switched to stably CAR modified T-cells to circumvent transient expression of CARs based on IVT-RNA. Thus, we decided to use virally transduced T-cells and target cells (Fig. 6A). Transduced T-cells were then evaluated for CAR expression (Fig. 6B), viability and TGFβRII KO efficiency. Real-time cytotoxicity was measured using the impedance based technique on an Xcelligence device using adherent CLDN6 + PA-1 Sc12 cell line as target and WT and KO TGFβRII CAR CLDN6- T-cells as effectors. Cells were incubated at an E:T ratio of 5:1.

WT and KO CAR CLDN6 T-cells were not meaningfully different in their cytotoxic function in the absence of TGFβ (Fig. 6C, red curve). However, in the presence of TGFβ, the killing efficacy of WT CAR CLDN6 T-cells was significantly impaired over the entire time range (72 h, black curve) to a steady-state level. Remarkably, although all three KO CAR CLDN6 T-cells revealed decreased cytotoxicity to the same extent within the first five hours after coculture setup with target cells, their cytolysis potency recovered in the presence of TGFβ and aligned with the level of cytolysis monitored over time for the non-TGFβ-treated controls (Fig. 6C/D) (% cytolysis was calculated based on the formula provided in Material and methods: Specific lysis was assured by subtracting cytolysis of mock T cells). The initial reduction could be attributed to an immediate suppressive effect of TGFβ captured here in real-time for bulk WT/KO T-cells against immunodeficient tumor cells in contrast to the results shown for immunostimulatory iDCs (Fig. 4C). After this lag-phase TGFβRII KO CAR T-cells became resistant to the TGFβ effect and caught up with non-TGFβ treated CAR T-cells in their cytolytic efficacy. Hence, we postulate that CAR T-cells can indeed be suppressed by TGFβ also in their cytotoxic function as resolved here in a real-time setting. Importantly, this can be reverted with TGFβRII KO CAR T-cells in the long run.

### Genomically edited TGFβ unresponsive CAR T-cells can escape control by induced regulatory T-cells.

**Fig. 7:**
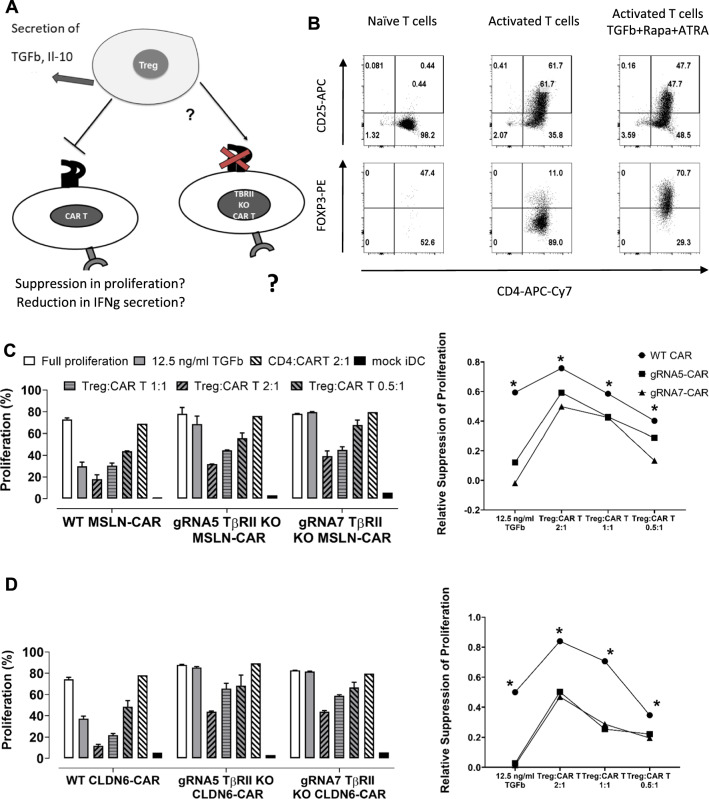
Genomically edited TGFβ CAR T-cells can escape control by induced regulatory T-cells. **A** Schematic representation of the working hypothesis to assess the inability of iTregs to suppress TGFβRII KO CAR T-cells: Genomic disruption of TGFβRII expression leads to functional unresponsiveness towards TGFβ secreted by iTregs ‘in situ’. This deactivates suppression of iTregs and hence, promotes better effector functions of responder T-cells such as antigen-dependent proliferation towards antigen electroporated iDCs. **B** Flow cytometry analysis of in vitro generated iTregs to confirm their phenotypic differentiation into CD4+ T-cells with regulatory functions. CD25 were overexpressed in both, CD4+ T-cells being solely activated by CD3 and CD28 and iTregs while the transcription factor FOXP3 was only overexpressed in the TGFβ/All trans retinoic acid /Rapamycine treated iTreg group. **C/D** Bar chart of normalized proliferation for WT and TGFβRII KO CAR T-cell groups, the latter genomically edited at the TGFβRII locus with 2 different gRNAs. CAR T-cell groups were either treated with exogenous TGFβ or cocultured with in vitro generated iTregs at iTreg:CART-ratios ranging from 2:1 down to 0.5:1. From this, iTregs can suppress WT CAR T-cell proliferation at any effector:responder-ratio in a dose-dependent manner. Bulk KO CAR T-cells are also prone to suppression by TGFβ secreted by iTregs, but are able to substantially resist, in particular at lower iTreg:CART-ratios (0.5:1 for CART MSLN; 0.5:1 and 1:1 for CART CLDN6) towards iDCs pulsed with MSLN (**C**) or CLDN6 (**D**). On the right, the relative changes in proliferation suppression are calculated from **C**/**D** left. P values were determined by two-way Anova using multiple comparison test. *P < 0.05; **P < 0.01; ***P < 0.001; ****P < 0.0001. In all experiments, mean ± SD of three technical replicates are given and experiments, involving T cells, are repeated for at least three donors

TGFβ not only commits naïve CD4 cells to differentiate into Tregs, but this cytokine also represents one of the principle mechanisms by which Tregs suppress their primary target cells, namely effector T-cells [28–30]. It has been shown in the past that T-cells which do not respond to TGFβ are able to escape control by regulatory T-cells [31]. It is well known that Tregs are highly abundant in TME, especially in tumors with high level of TGFβ secretion, so we prompted us to verify whether TGFβRII KO CAR T-cells were not only able to withstand TGFβ but also to resist the suppressive effect of Tregs (Fig. 7A). For such experiments, we were challenged to generate Tregs ex vivo from PBMCs in sufficient numbers which were referred to as induced regulatory T-cells (iTregs). The detailed protocol for generating iTregs has been described elsewhere [32]. In brief, CD4+ cells were activated with anti CD3, anti CD28 and IL2. The CD4+ cells which were treated this way represent activated CD4+ T-cells as negative control for immunosuppression. For differentiation into iTregs, rapamycin, retinoic acid and TGFβ were subsequently added to the culture medium. T-cells were activated for up to 6 days, and then analyzed for FOXP3 and CD25 expression to confirm their iTreg phenotype (Fig. 7B). One day before setting up the coculture WT and TGFβRII KO T-cells were electroporated with CAR MSLN (Fig. 7C) or CLDN6 (Fig. 7D) IVT-RNAs and iDCs were electroporated with the respective antigen encoding IVT-RNAs. Since we aimed at demonstrating cessation of the suppressive effect of induced regulatory effector T-cells in a proliferation assay, responder CD8+T-cells were stained with CFSE before seeding. For setting up the coculture, fixed numbers of iDCs and WT or KO CAR T-cells were coseeded and iTregs were added for different ratios to CAR T-cells (2:1, 1:1 and 0.5:1; Fig. 7C/D). The responder groups comprised beside the WT CAR T-cells as reference 2 different TGFβRII KO CAR Tcell groups each treated with one of the CRISPR/Cas9 gRNAs 5 or 7, respectively. As positive control we included the addition of exogenous TGFβ in the absence of iTregs, to confirm on the one hand that WT CAR T-cells are susceptible to suppression and on the other hand that TGFβRII KO CAR T-cells generated ex vivo are resistant to the anti-proliferative effect of exogenous TGFβ (Fig. 7C/D, second outmost left). Eventually, WT and KO CAR T-cells elicited comparable rates of proliferation in the absence of exogenous TGFβ (Fig. 7C, D outmost left). Activated CD4+ T-cells turned out to be not able to suppress WT and TGFβRII KO CAR T-cells. Intriguingly, in vitro generated iTregs can inhibit proliferation of WT CAR T-cells even at the lowest iTreg to CAR T-cell ratio, ranging from excess 2:1 down to 0.5:1. Most importantly, although iTregs can also inhibit the TGFβRII KO CAR T-cells a little, the suppressive effect is less pronounced in a iTreg dose-dependent fashion compared to that in case of WT CAR T-cell. The remaining suppressive effect in 2 gRNA treated groups may at least in part be explained by the bulk T-cell populations used here, comprising unedited and hence TGFβ-responsive WT CAR T-cells and TGFβRII KO CAR T-cells in each group. We should also keep in mind that Treg exert several other mechanisms to suppress T cells such as secretion of IL-10 and IL-35 [33] and hence the TGFβRII KO CAR T cells are not fully resistant to iTregs. This phenomena could be a layer of safety in using these kind of KO CAR T cells as they are not fully resistant to regulatory mechanisms by the immune system. The suppressive effect is most likely due to TGFβ produced endogenously by the induced regulatory T-cells leading to a paracrine effect because we solely targeted TGFβRII via CRISPR/Cas9 in responder T-cells. Since iTregs have not been equipped with CLDN6 or MSLN-specific CARs, iTreg/iDC*Ag cell/cell contacts are highly unlikely to occur and to contribute to the suppressive effect towards responder T-cells observed here [34].

### *TGFβRII* KO rescues CAR T-cell exhaustion induced by TGFβ

Finally, we explored if TGFβ is also able to foster CAR T-cell exhaustion characterized by decreasing functional responsiveness, using a repetitive antigen stimulation assay, and whether the KO of TGFβRII on CAR T-cells are able to prevent this counter-regulatory effect. In a series of experiments, we took advantage of the impedance based cell killing assay: For this, the generation of stably modified CAR T-cells by means of retroviral transduction was a prerequisite in long term restimulation experiments. CAR T-cells were cocultured with the cell line OCVAR3 which expresses CLDN6 endogenously, at an E:T ratio of 5:1.

**Fig. 8:**
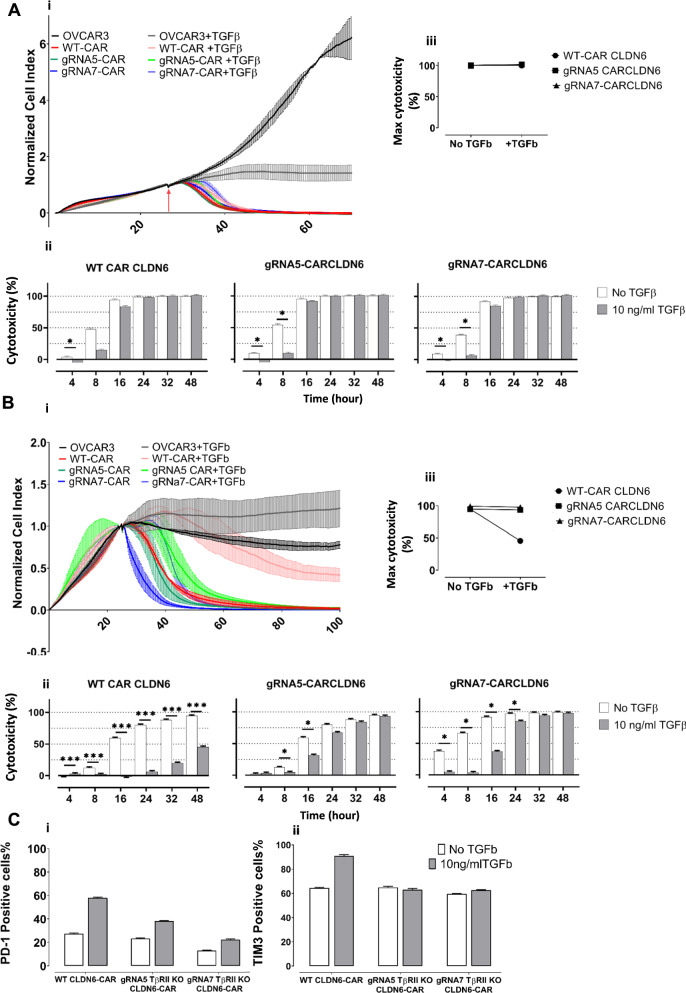
*TGFβRII* KO counteracts CAR T-cell exhaustion induced by TGFβ. **A** OVCAR-3 cell line were seeded on an xcelligence plate and after 20 h, retrovirally transduced WT or TGFβRII KO CAR CLDN6 T-cells were added at an E:T ratio of 5:1 in the presence or absence of TGFβ. After 48 h, T-cells were collected, washed and counted, and seeded again on a new plate with freshly seeded adherent OVCAR-3 cell line almost growing in saturation phase (20 h). This procedure was repeated one more time to end up with 3 cycles of antigen-specific stimulation. i) A first round of coculture demonstrated that normalized cell indices for WT and TGFβRII KO CAR T-cells were barely affected by the presence of TGFβ; ii) a first round of coculture did also not hamper cytotoxicity of any CAR T-cell group. iii) Max cytotoxicity is given here for a late time point (48 h of coculture) indicating no difference in cytotoxic efficacy. **B** A third round of coculture demonstrated a vast effect of TGFβ on cytotoxicity of WT CAR CLDN6 T-cells**. **i) Normalized cell indexes for third round of coculture of WT CAR T-cells or TGFβRIIKO with OVCAR-3 cells indicate a significant difference in cytotoxicity when comparing these groups. ii) Bar chart of the calculated cytotoxicity demonstrating a long-lasting repression of WT CAR CLDN6 T-cells while the TGFβRII KO CAR T-cells recovered from a short suppression phase and remained fully functional in the end (48 h). iii) Max cytotoxicity is given here for a late time point (48 h of coculture) indicating a substantial difference in cytotoxic efficacy. P values were determined by two-way Anova using multiple comparison test. *P < 0.05; **P < 0.01; ***P < 0.001; ****P < 0.0001. In all experiments, mean ± SD of three technical replicates are given and experiments, involving T cells, are repeated for at least three donors. **C** After third round of coculture with OVCAR-3, the CAR T cells were collected, washed and stained for immune check point and exhaustion markers PD-1 and TIM 3. Bar chart representing the percentage of i: PD-1 and 2: TIM3 positive cells are shown

In the first (Fig. 8Ai) and second (data not shown) round of stimulation TGFβ revealed initial suppression within a few hours (Fig. 8Aii/Aiii) on cytotoxicity, but was not able to mount a long-lasting suppressive effect on the cytotoxicity of the CAR T-cells, neither on WT nor TGFβRII KO CAR T-cells, the latter shown for gRNA 5 and 7, respectively. The reduction in cytotoxicity in TGFβ-treated samples was roughly the same for WT and TGFβRII KO CAR T-cells. Between 8 and 16 h of coculture lysis became complete for both groups. In the course of the third round of coculture (Fig. 8Bi), TGFβ-untreated WT and KO T-cell groups sustained cytolytic function over time. On the other hand, WT CAR T-cells turned out to be for the first time vastly impaired in their cytotoxic function in the presence of TGFβ over the whole time frame (Fig. 8Bii): 48 h after setting up the assay lysis dropped down to less than 50% in comparison to the TGFβ untreated group.

In contrast, TGFβ-treated TGFβRII KO CAR T-cells recovered from a short suppression phase within 16 h and retained full lysis potency comparable to their related TGFβ-untreated groups lasting till the end of experiment (48 h). We also checked the expression of two important exhaustion markers, PD-1 and TIM3 on the surface of different T cell groups after the third antigen stimulation. As it is depicted in Fig. 7C, all groups, upregulated the expression of this markers upon exposure to TGFβ, however the upregulation in case of wild type CAR T cells was much more pronounced which is in line with our cytoxicity data.

We reasoned to see a suppressive effect of TGFβ on WT CAR T-cells only in round 3 of stimulation because the moderate endogenous expression rate of CLDN6 on ovarian cancer c.l. OVCAR-3 prevents premature exhaustion of WT CAR T-cells. This contrasts to the much higher expression seen on teratoma c.l. PA1, the latter which promotes early differentiation into senescent effector T-cells and hence, sensitivity towards TGFβ of WT CAR T-cells already after initial antigen-specific stimulation as outlined in Fig. 6.

To sum up the restimulation experiment, TGFβ exerts a less pronounced prohibitive effect on freshly activated CAR T-cells irrespective of being genomically silenced for TGFβRII expression or not, but affects significantly cytotoxicity of repetitively antigen-experienced, and hence senescent CAR T-cells. Knock out of the TGFβRII via CRISPR/Cas9 turns CAR T-cells, after a short lag-phase, into effector T-cells unresponsive towards TGFβ in the long term and hence, emphasizes the benefit of ourapproach in generating more potent long-lived CAR T-cells in immunosuppressive TME.

## Discussion

TGFβ is highly produced by many tumor types including ovarian malignancies [35, 36]. Tumors can promote TGFβ production by tumor microenvironment resident cells such as fibroblasts [37, 38]. TGFβ is a pleiotropic cytokine with diverse effects on cancer cells and the immune system. In premature phases of tumorigenesis, TGFβ acts as a tumor suppressor [39], whereas during progressed tumor immunoediting [40] cancer cells become resistant to the signaling and antitumor activity of this cytokine and in late stages, they gain genomic mutations which turn TGFβ to a tumor promoting agent [7, 41]. On the other hand, TGFβ is a key regulator of immune homeostasis; in the context of T cells, TGFβ can inhibit proliferation, expression of cytolytic gene cytokines such as IFN-γ and Granzyme B and Th1 differentiation and at the same time has a pivotal role in Treg generation [5, 10, 24]. There are also several reports on TGFβ suppressive effects on CAR T-cells functions, in terms of proliferation, cytotoxicity and cytokine secretion [9, 23, 25, 42].

Having in mind that TGFβ is likely to be one of the most important suppressor cytokines of CAR T-cell function, one may take into consideration different approaches to block its effects. Among the alternatives, localized blockade of TGFβ in tumors is not the best option, since tumor cells are not the only source of TGFβ in TME [5]. Although systemic blockade of TGFβ administering small-molecules or antibodies may act in a more efficient way it couldn’t be ruled out to put the patient at risk of autoimmune disease. It should also be noted that TGFβ triggers apoptosis in pre-malignancies, so the systemic inhibition of TGFβ is not a reasonable option as it may confer tumorigenesis in other tissue regions [7].

Among the different possibilities, inhibiting the TGFβ signaling pathway in T-cells per se is an intriguing idea. Recently, a novel TGFβ specific CAR harboring a scFv against TGFβ has been reported which has the capacity of inhibiting endogenous TGFβ signaling and more interestingly converting TGFβ into a stimulus of T-cell growth [20]. Several other groups also attempt different approaches to tailor TGFβ pathway in the context of CAR T-cell therapy so as to improve CAR T-cell function in solid tumors.

Since TGFβRII is the first cellular component in TGFβ signaling, it is reasonable to target this receptor in the first place to block immunosuppressive T-cell signaling. Kloss et al. reported on the overexpression of a dominant negative TGFβRII which lacks the intracellular signaling domain in PSMA specific CAR T-cells leading to improved CAR T-cell function in vitro and in vivo [9]. However, this approach still allows for the expression of the wild type TGFβRII and hence, will not abolish TGFβ-mediated signaling in T-cells. Tang et al. reported that knocking out the endogenous TGFβRII in CAR T-cells could reduce conversion to induced Tregs and prevent the exhaustion of CAR T-cells and led to better in vivo tumor elimination efficacy of CD28 second generation CARs specific for MSLN [25]. However, the authors did not assess, whether TGFβ produced by Tregs endogenously will affect other T-cells’ function, as we could confirm in our work here. In a very recent study, Liu et al. [43] using a Cre-lox system, demonstrated that ablation of TGFβRII in CD4+ T-cells promotes tumor tissue healing and halts cancer progression, while in a back to back publication Li et al. [44] showed that blocking TGFβ signaling in CD4+ T-cells via Cre-lox system remodels the tumor microenvironment and restrains cancer progression. These approaches thoroughly demonstrate the usefulness to target TGFβRII-signaling to foster T-cell effector functions in several models. However, they did not preclinically assess CRISPR/Cas9 to disrupt TGFβRII as one of the most advanced tool in genomic editing, which already entered clinical phases in other studies [45]. Here, we hypothesized that genomically knocking out the TGFβ receptor via CRISPR/Cas9 in CAR T-cells will improve their functions in a solid tumor context as they will be able to resist the inhibitory effect of TGFβ in the immunosuppressive TME.

One of the most important obstacle is the choice of an optimal antigen [46–48]: Beside lack of tumor specific antigens on tumor cells, the majority of CAR T-cells targeting solid tumors are based on tumor associated antigens, which are highly expressed on tumor cells, but at the same time to some extent on normal tissue, which may lead to ‘on target’ but ‘off tumor’ toxicities. Here, we focused on two ovarian cancer associated antigens which were highly likely not or very rarely expressed on normal tissues. Reinhard et al. draw recently the conclusion that CLDN6 is an oncofetal cell-surface antigen with an ideal expression profile for CAR T-cell mediated immunotherapy (Fig. 1A) [13]. To also generalize our strategy we also focused on mesothelin (MSLN) specific CAR T-cells, which has been shown to represent a safe target in several clinical studies [49].

One should keep in mind that despite having CRISPR/Cas9 KO efficacies in the range of 20–60% in CD4+ and CD8+ T-cells depending on the gRNA used, we conducted our experiments with bulk CAR T-cells encompassing edited and unedited cells. Thus, the beneficial effects in TGFβ resistance should be even more pronounced for homogenously edited or purified T-cell populations. Nonetheless, we could clearly demonstrate that CAR T-cells benefit from knocking out TGFβRII in different aspects of anti-tumor function:

First, genomic inactivation of the TGFβRII signaling pathway does not affect expression of exogenously introduced CAR molecules irrespective of the expression system, which is an essential prerequisite to generate antigen-specific effector T-cells.

Secondly, TGFβRII KO CAR T-cells were able to resist the anti-proliferative effect of TGFβ even at the highest dose of anti-inflammatory cytokine provided. This may not only positively affect the differentiation status and hence, viability and persistence of CAR T-cells but most importantly, the absolute number of therapeutic T-cells at the tumor site: Tumor eradication turns out to be the more efficient the more potent effector cells of a non-senescent phenotype such as central memory T-cells (T_CM_) mount an immune response against tumor cells [50]. Moreover, we demonstrated that responder TGFβRII KO CAR T-cells were also able to proliferate in the presence of TGFβ secreted ‘in situ’ by regulatory T-cells, which mimicks, at least in part, the immunosuppressive milieu in TME.

Thirdly, CAR T-cells are able to rescue their cytotoxic function in the presence of exogenous TGFβ, even under conditions of repetitive antigen exposures: Here, we show that CD8+ TGFβRII KO CAR T-cells retain their primary function in the presence of exogenously added excess TGFβ. Antigen-experienced TGFβRII KO CAR T-cells withstand suppression triggered by TGFβ and proved to retrieve full lysis upon successive cycles of restimulation despite their advanced differentiation in direction of a senescent phenotype. This feature is key to maintain a long-lived cytotoxic T-cell population to prevent the outgrowth of clonal tumor escape variants.

Fourthly, TGFβRII KO CAR T-cells were also able to preserve a set of cytokine secretions, whereas WT CAR T-cells were substantially hindered in secreting the proinflammatory cytokines such as TNF-α and IFNγ, the growth factors GM-CSF and IL2, and the cytotoxic mediator Granzyme B in the presence of exogenous TGFβ scrutinized here. In summary, our results support the notion that blocking the TGFβRII signaling pathway by means of its genomic deletion via CRISPR/Cas9 may represent a novel approach to antigen-specifically unleash anti-tumor CAR T-cell function in an otherwise suppressive tumor microenvironment.

## Conclusion

It has been well established that TGFβ significantly suppresses the cytotoxic function of CD8+ T cells and also affects CD4+ T-cell differentiation and function by inducing their conversion into regulatory T-cells.

In the current study we prompted us to investigate the effect of a shutdown of the TGFβ signaling pathway exclusively in T-cells for two different second-generation BBς CARs specific for MSLN and CLDN6, respectively, taking advantage of the CRISPR/Cas9 genome editing tool. Our study clearly demonstrated that CAR T-cells benefit form knocking out TGFβRII in different aspects of anti-tumor functionality: TGFβRII KO CAR T-cells could resist the anti-proliferative effect of TGFβ even at the highest dose of cytokine applied, and they could preserve their cytotoxic function in the presence of exogenous TGFβ even in repetitive antigen exposure experiments. They could also retain their cytokine secretion profile, comprising TNF-α, IFNγ, GM-CSF, IL2, and Granzyme B. We could also demonstrate that TGFβRIIKO CAR T-cells can withstand the TGFβ-mediated suppressive effect of iTregs ‘in situ’. In contrast, for WT CAR T-cells all effector functions assessed here were significantly impaired in the presence of TGFβ. These observations may emphasize the perspective to apply the genomic disruption of the TGFβRII-mediated signaling pathway as a means of fostering efficacy of CAR T-cell immunotherapy against immunosuppressive tumor entities in future clinical trials.

## Supplementary Information

**Additional file 1: Figure S1.** SK-OV-3 spheroid killing assay demonstrated that CAR MSLN T-cells react specifically and while recognize and kill HeLa cells at very low E:T ratio of 1:1, does not kill the SK-OV-3 cells at E:T ratio of 30:1.

**Additional file 2: Figure S2.** Histogram sequencing results for wild type and A: gRNA3 and B: gRNA5 treated groups, elicit a heterologous sequence 4 bases inside the gRNA target target sequence upstream of complementary 5’-NGG PAM-sequence, which is in line with indel formation.

**Additional file 3: Figure S3.** Genomic TGFβ RII disruption in CAR T-cells specific for CLDN6 enhances their cytokine secretion. TGFβ at any dose inhibits cytokine secretion of CLDN6-specific WT CAR 4-1BBζ T-cells. A: IL-2, B: IFNγ, C: TNFα, D: GM-CSF, E: Granzyme B, in the presence of antigen presenting iDCs as APCs. Of importance, amount of cytokines in TGFβRII KO CAR T-cells remain unimpaired and stable even at a very high dose of exogenous TGFβ. Cytokine secretion in either WT or KO groups proved to be antigen specific here as well. P values were determined by two-way Anova using multiple comparison test. *P < 0.05; **P < 0.01; ***P < 0.001; ****P < 0.0001. In all experiments, mean ± SD of three technical replicates are given and experiments, involving T cells, are repeated for at least three donors.

## Data Availability

The datasets used and analyzed during the current study are available from the corresponding author on reasonable request.

## Abbreviations

CAR T-cells: Chimeric antigen receptor T-cells
c.l.: Cell line
TGFβ: Transforming growth factor β
TGFβRII KO CAR T-cells: TGFβ receptor II knocked out CAR T-cells
IVT: In vitro transcribed
MSLN: Mesothelin
CLDN6: Claudin 6
