# Supplementary figures and images for: CRISPR/Cas9-mediated TGFβRII disruption enhances anti-tumor efficacy of human chimeric antigen receptor T cells in vitro
Source: J Transl Med. 2021 Nov 27;19:482. doi: 10.1186/s12967-021-03146-0 (PMC8627098; doi:10.1186/s12967-021-03146-0)

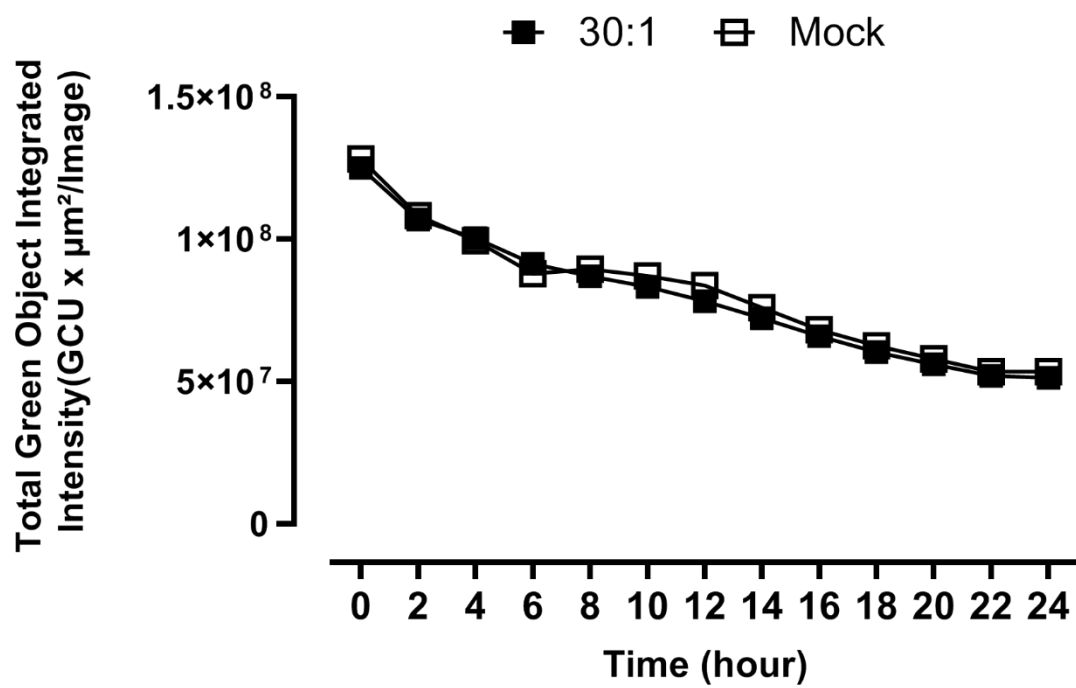

Supplement: Supplementary file 1 — Additional file 1: Figure S1. SK-OV-3 spheroid killing assay demonstrated that CAR MSLN T-cells react specifically and while recognize and kill HeLa cells at very low E:T ratio of 1:1, does not kill the SK-OV-3 cells at E:T ratio of 30:1. [file 12967_2021_3146_MOESM1_ESM.pdf]

**A**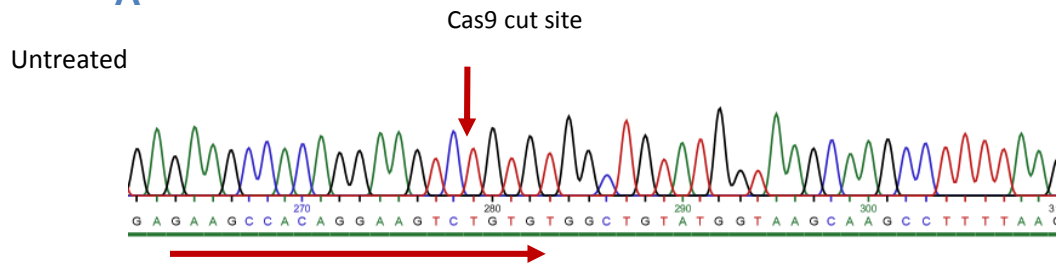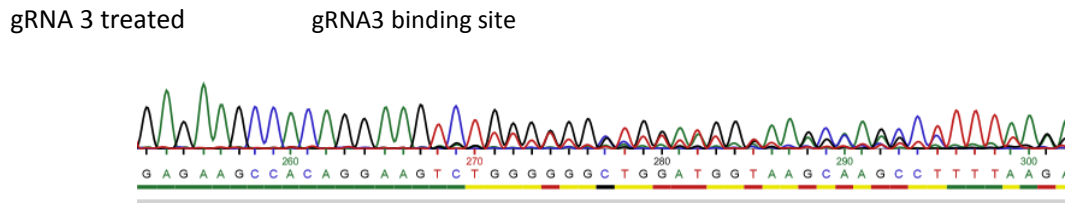**B**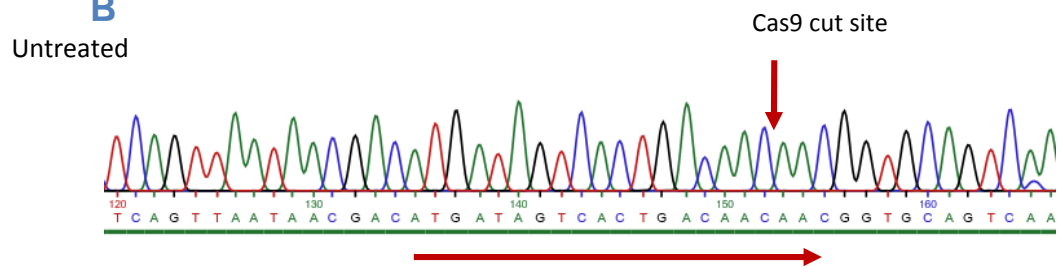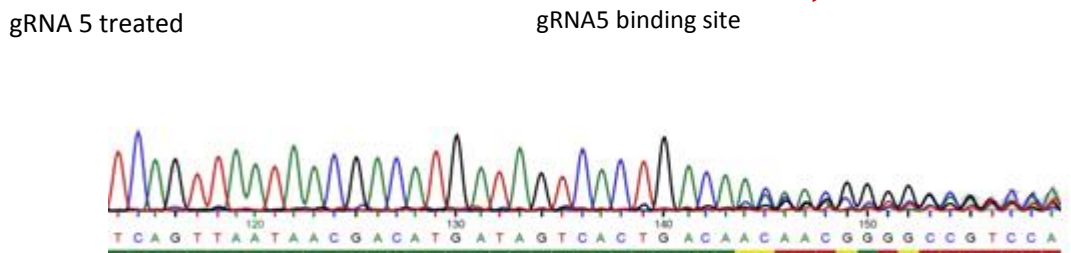

Supplement: Supplementary file 2 — Additional file 2: Figure S2. Histogram sequencing results for wild type and A: gRNA3 and B: gRNA5 treated groups, elicit a heterologous sequence 4 bases inside the gRNA target target sequence upstream of complementary 5’-NGG PAM-sequence, which is in line with indel formation. [file 12967_2021_3146_MOESM2_ESM.pdf]

**A**

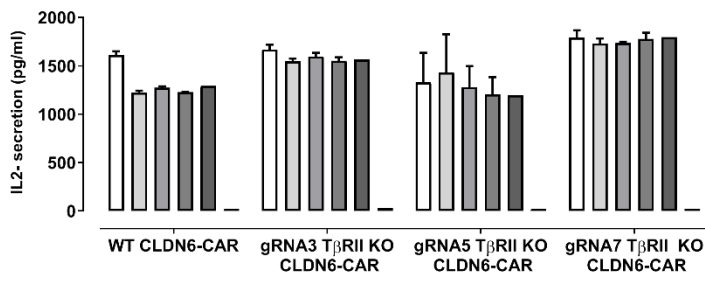

**B**

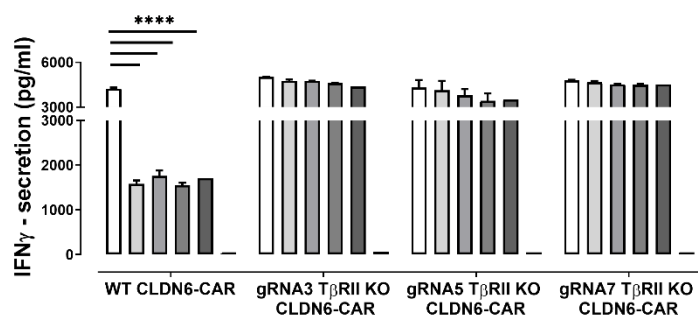

**C**

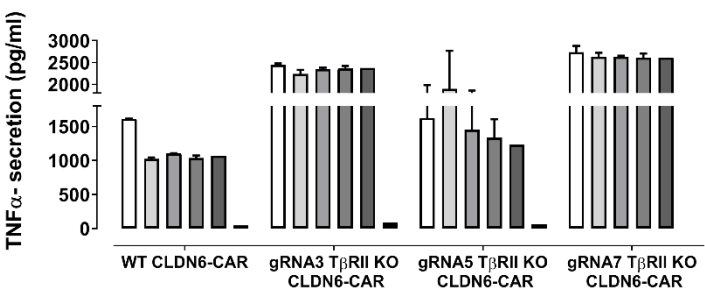

**D**

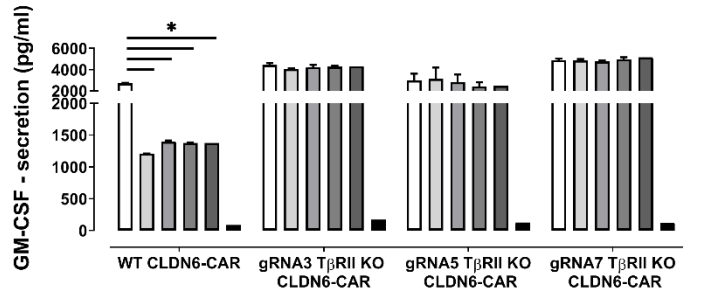

**E**

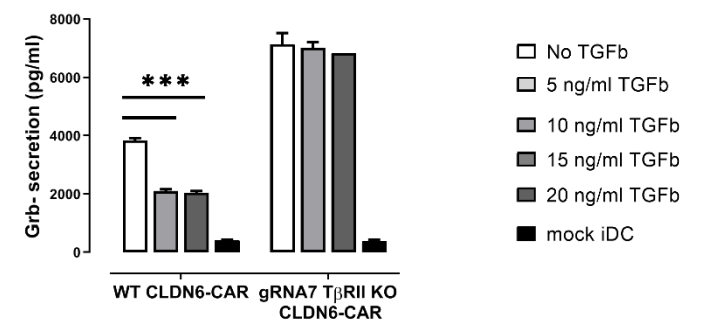

Supplement: Supplementary file 3 — Additional file 3: Figure S3. Genomic TGFβ RII disruption in CAR T-cells specific for CLDN6 enhances their cytokine secretion. TGFβ at any dose inhibits cytokine secretion of CLDN6-specific WT CAR 4-1BBζ T-cells. A: IL-2, B: IFNγ, C: TNFα, D: GM-CSF, E: Granzyme B, in the presence of antigen presenting iDCs as APCs. Of importance, amount of cytokines in TGFβRII KO CAR T-cells remain unimpaired and stable even at a very high dose of exogenous TGFβ. Cytokine secretion in either WT or KO groups proved to be antigen specific here as well. P values were determined by two-way Anova using multiple comparison test. *P < 0.05; **P < 0.01; ***P < 0.001; ****P < 0.0001. In all experiments, mean ± SD of three technical replicates are given and experiments, involving T cells, are repeated for at least three donors. [file 12967_2021_3146_MOESM3_ESM.pdf]
